# Raptured gas forming pyogenic liver abscess with a biliary fistula in Botswana: a case report

**DOI:** 10.11604/pamj.2023.46.107.42256

**Published:** 2023-12-18

**Authors:** Andrew Ojuka, Chiapo Lesetedi, Christian Jabo, Kgosidialwa Frederick Mompati

**Affiliations:** 1 Riverside Hospital, Francistown, Botswana; 2 Bokamoso Private Hospital, Gaborone, Botswana

**Keywords:** Gas-forming liver abscess, pyogenic liver abscess, biliary fistula, Botswana, case report

## Abstract

We report a case of gas-forming pyogenic liver abscess (GFPLA) with a ruptured abscess and biliary fistula presenting with peritonitis. The patient had poorly controlled diabetes mellitus and was extremely ill at presentation. The diagnosis was delayed until the abscess ruptured, owing to nonspecific abdominal symptoms at the initial presentation and delayed follow-up radiological investigations. The patient had a high-output biliary fistula post-operatively, which was managed with endoscopic retrograde cholangiopancreatography (ERCP) and stenting with fistula drainage reduction within four weeks. This case highlights the need for follow-up radiological investigations and prompt intervention in patients with diabetes mellitus presenting with fever and vague abdominal pain.

## Introduction

Gas-forming pyogenic liver abscess (GFPLA) is rare and is associated with a high mortality rate [1]. It is commonly associated with underlying diabetes mellitus (DM) [1,2]. Gas formation occurs because of mixed acid fermentation within the abscess by formic hydrogenlyase, an enzyme produced by some bacteria [2,3]. The presentation can be nonspecific, leading to a delayed diagnosis [4]. Management includes urgent abscess drainage, either percutaneously or surgically [1,3]. A biliary fistula can be a complication if the abscess cavity is large and erodes into intrahepatic biliary ducts. It is best managed endoscopically by ERCP and stenting [5]. Limited data and reports on GFPLA are available in sub-Saharan Africa and only a few cases have been reported worldwide.

## Patient and observation

**Patient information:** we present a 39-year-old male diabetic who was referred from a public hospital with peritonitis that required urgent surgical intervention. He was unwell for approximately four weeks with a history of abdominal pain in the epigastrium, low-grade fever, nausea, and vomiting but no diarrhea. The abdominal ultrasound scan performed initially at the onset of his symptoms was unremarkable. Before referral, he was admitted to a public hospital, where he received treatment for approximately 15 days. He did not improve, and 4 days before referral, he progressed to develop generalized abdominal pain that started in the right hypochondrium.

**Clinical findings:** examination on arrival revealed a sick patient who was dehydrated with respiratory distress, with a temperature of 36.4°C, pulse rate of 93 bpm, and a blood pressure of 145/90 mmHg. The abdomen was moderately distended with generalized tenderness and guarding was more marked in the suprapubic and right hypochondrial areas. The frequency and pitch of the bowel sounds were reduced. The blood sugar was 7.5 mmol/l and HbA_1_C was 10.02%. There was leucocytosis (12.5 x10^3^/ul) predominately neutrophilia (10x10^3^/ul), moderate anemia (8.0 g/dl), raised liver enzymes S-ALP (670.9u/l), S-AST (98.4u/l), S-ALT (52.6u/l), S-GGT (362.1u/l). The total bilirubin level was elevated (32.9u/l), and the direct bilirubin was (18.2u/l). He had deranged renal function, S-eGFR (40.25 ml/min/1.73 m^2^), S-Urea (10.07 mmol/l), and S-creatinine (161.4ummol/l). Widal test results were negative and stool analysis was unremarkable. Serology for entamoeba histolytica and echinococcus granulosus antibodies was negative.

**Timeline:** the patient was referred and admitted to our unit on 30^th^ September 2021. Exploratory laparotomy was done on 30^th^ September 2021. Transfer for ERCP was on 6^th^ December 2021. Hospital discharge on 28^th^ December 2021. The patient died in mid-January 2022. The planned stent removal was on 27^th^ January 2022.

**Figure 1:**
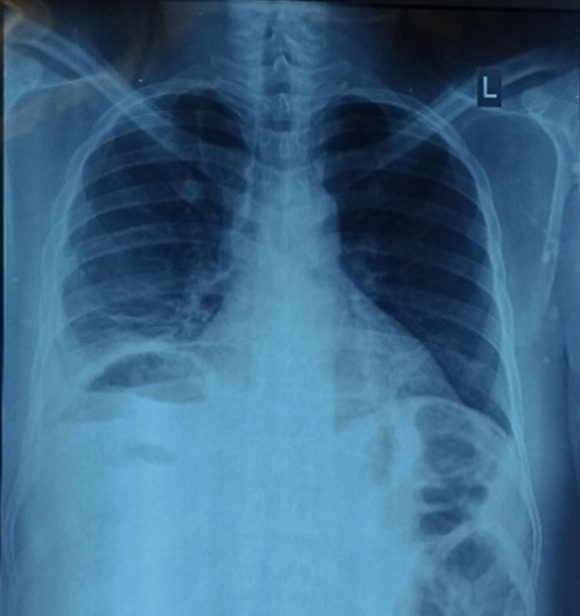
posterior anterior chest X-ray showing the air-fluid level below the right hemidiaphragm

**Figure 2:**
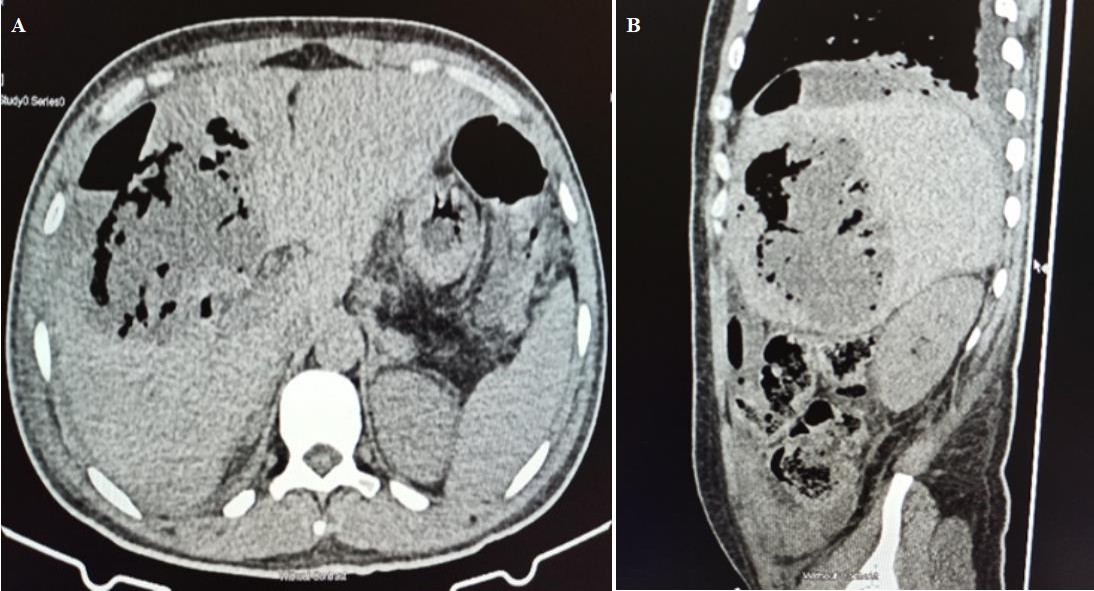
A,B) axial and sagittal non-contrast computed tomography scan of the abdomen showing a large gas-forming liver abscess in segments VII and VIII with peri-hepatic and intrahepatic air-fluid levels

**Diagnostic assessment:** chest radiograph revealed an air-fluid level under the right hemidiaphragm (Figure 1). Non-contrasted abdominopelvic computed tomography (CT) scan showed a large (14 x 12 x 9 cm) abscess in segments IV, VII, and VIII of the liver, intrahepatic air-fluid levels, hydropneumoperitonium but no stones in the biliary tree (Figure 2). Contrast could not be given because of the patient´s deranged renal functions.

**Diagnosis:** a ruptured gas-forming pyogenic liver abscess was diagnosed, and a differential diagnosis of perforated viscus was made.

**Prognosis:** the prognosis was equivocal considering the delayed diagnosis and diagnostic findings.

**Figure 3:**
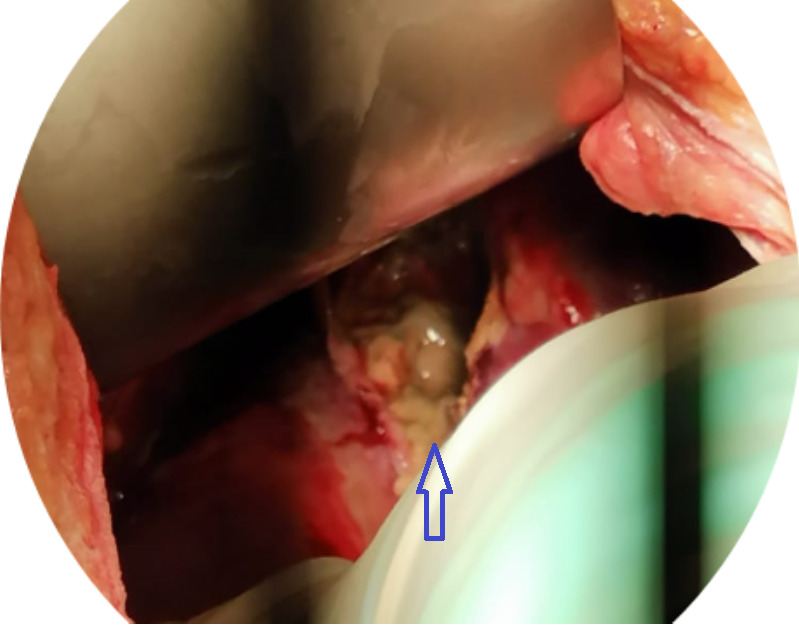
the area pointed by the arrow showing the abscess cavity inside the liver parenchyma that was exposed after the debridement of the necrotic roof of the liver

**Therapeutic interventions:** the patient underwent an exploratory laparotomy. During surgery, 400 mL of bilious fluid was found in the peritoneum, a walled-off pelvic abscess with 300 mL of frank pus, walled-off right subphrenic gas, and an abscess with 800 mL of frank pus, grade 1 small bowel adhesions, and liver capsule necrosis (6 x 6 cm) in segments VII and VIII, which ruptured and drained a mixture of pus and bile from the abscess cavity (Figure 3). There was no bowel perforation and the gall bladder was normal. The abscesses were drained and debridement of the necrotic area on the liver surface was performed. The greater omentum was tagged into the abscess cavity, followed by adhesion lysis and peritoneal lavage. Two closed abdominal drains were placed in the right subphrenic and suprapubic regions.

**Figure 4:**
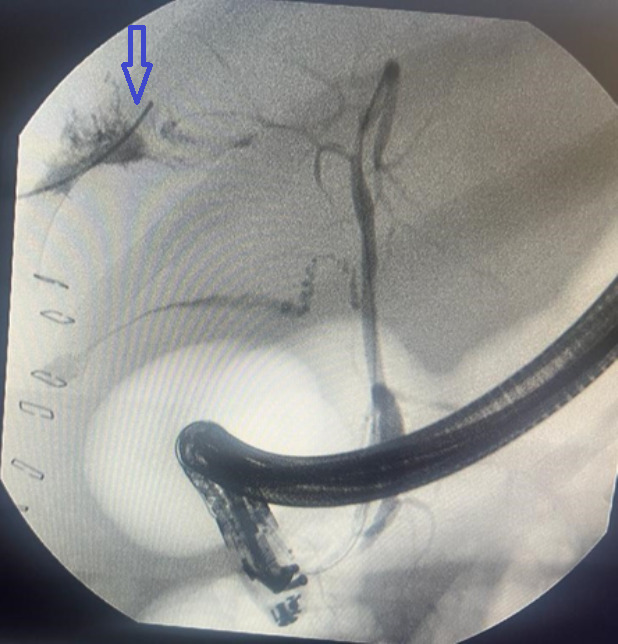
demonstrating endoscopic retrograde cholangiopancreatography with spillage of contrast through the fistula (arrow)

**Follow-up and outcome of interventions:** postoperatively, the patient was admitted to the intensive care unit (ICU) with ceftriaxone and metronidazole antibiotics. He was transfused with three units of whole blood. Insulin was administered and ventilated for 2 days before extubation. On the third post-operative day, he was able to tolerate liquids. The abdominal pus swab yielded no organisms on microscopy or culture. The patient was stable on the 6^th^ postoperative day, with a normal white blood count (5.45 x 10^3^/ul). However, there was still significant daily drainage of approximately 600 mL of bilious fluid from the abdominal drain, confirming the complication of a biliary fistula. The patient was transferred for ERCP and stenting because the service was unavailable at our center. The procedure was uneventful. No common bile duct (CBD) stones were observed (Figure 4).

After CBD stenting, the drainage of the bilious fluid was reduced to approximately 20 mL/day and eventually stopped. The patient was discharged after four weeks, with planned stent removal within six weeks. However, before reviewing for stent removal, he developed severe hypoglycemia. He was admitted to the primary hospital close to his home, where he, unfortunately, passed on after two days with DM complications.

**Patient’s perspective:** the patient was happy with the treatment outcome before he was discharged from the hospital.

**Informed consent:** it was obtained from the patient.

## Discussion

Gas-forming pyogenic liver abscess (GFPLA) is a rare condition, and there is limited data on GFPLA in Botswana and Southern Africa. A study reviewing the current etiology and treatment of pyogenic liver abscess (PLA) in 105 patients in a South African teaching hospital by Chihaka *et al*. reported biliary sepsis as the leading cause of PLA [3]. They reported *Klebsiella pneumoniae* and *Escherichia coli* were the predominant organisms cultured [2]. *Klebsiella pneumoniae* and *Escherichia coli* are known to cause GFPLA in diabetic patients [1,3]. Other organisms that cause GFPLA include *Salmonella spp* and *Clostridium spp* [1,3]. In diabetic patients with poor blood sugar control, the bacterial fermentation of glucose produces formic acid. At an acidic PH (<6), bacterial formic hydrogenlyase converts formic acid to carbon dioxide and hydrogen gases [2,4]. Our patient had gas formation in the liver, though, we could not isolate any organism from the culture. This could have been due to the antibiotic treatment administered before referral. Common presentations include fever and abdominal pain though symptoms can be nonspecific resulting in a delay in diagnosis [4].

The diagnosis for our patient was delayed because of nonspecific symptoms at the onset of his condition and a negative initial abdominal ultrasound scan. This contributed to the delayed decision to perform an abdominopelvic CT scan. It is possible that an ultrasound scan was performed when the abscess had not yet formed or was missed. Diagnosis can easily be made with radiological imaging. The sensitivities for diagnosing pyogenic liver abscess using an abdominal ultrasound scan and abdominal computer tomography are 85% and 97% respectively [6]. Radiographs may show pockets of gas within the right hypochondrial soft tissue shadow (liver shadow), but this has been reported to be visible in only up to 36% of patients with liver abscesses [2]. It is dependent on the amount of gas accumulated and, unless suspected, may be mistaken for bowel gas [2]. Patients with GFPLA are sick and disease progression can be very rapid [7,8]. Two large case series studies from Taiwan involving 28 and 83 patients with GFPLA showed significant differences between GFPLA and non-GFPLA [7,8]. These studies showed a statistically higher incidence of septic shock, bacteremia, and mortality in patients with GFPLA compared to non-GFPLA patients [7,8]. Symptom duration was also shorter with more abnormal blood parameters, such as higher serum glucose, urea, aspartate transaminase, aminotransferase, and alkaline phosphatase [7]. Our patient also had deranged liver and renal functions and required post-operative admission to the ICU.

The management of GFPLA includes hemodynamic support, broad-spectrum intravenous antibiotics, and urgent drainage, which can be either percutaneous or surgical because the risk of rupture is high [9,10]. Surgical drainage is very effective, particularly for large and ruptured abscesses [9,10]. Poor microcirculation in affected areas has also been postulated to contribute to gas accumulation [2,4]. This may explain the higher incidence of GFPLA in patients with diabetes mellitus [8,10]. Our patient had poorly controlled diabetes mellitus with an HbA1C level of 10.02%, which could have facilitated gas formation in the abscess.

A biliary fistula can be a complication of pyogenic liver abscess if the abscess cavity is large and erodes into the intrahepatic ducts [5]. It is best managed endoscopically with ERCP and stenting, with a mean healing time of 6 days in some studies (range 4-40 days) [5]. Endoscopic techniques reduce the pressure gradient between the bile duct and duodenum, maintained by an intact sphincter of Oddi, and divert the bile away from the leak site, resulting in fistula closure [5]. Our patient underwent ERCP and stenting (Figure 4), and the fistula output significantly reduced from 600 mL/day to less than 20 mL/day over 48 h, with healing within 4 weeks.

## Conclusion

To reduce fatality from GFPLA, immediate radiological and clinical diagnosis, control of blood sugar levels, and early percutaneous or surgical management are essential. It is important to have dedicated intensive care during the post-operative period for optimal outcomes, and early ERCP and stenting for biliary fistula management.
